# Epistasis of Transcriptomes Reveals Synergism between Transcriptional Activators Hnf1α and Hnf4α

**DOI:** 10.1371/journal.pgen.1000970

**Published:** 2010-05-27

**Authors:** Sylvia F. Boj, Dimitri Petrov, Jorge Ferrer

**Affiliations:** 1 Genomic Programming of Beta-Cells Laboratory, Institut d'Investigacions Biomèdiques August Pi i Sunyer, Barcelona, Spain; 2 Endocrinology Department, Hospital Clínic de Barcelona, Barcelona, Spain; 3 Centro de Investigación Biomédica en Red de Diabetes y Enfermedades Metabólicas Asociadas, Barcelona, Spain; Universtity of Pennsylvania, United States of America

## Abstract

The transcription of individual genes is determined by combinatorial interactions between DNA–binding transcription factors. The current challenge is to understand how such combinatorial interactions regulate broad genetic programs that underlie cellular functions and disease. The transcription factors Hnf1α and Hnf4α control pancreatic islet β-cell function and growth, and mutations in their genes cause closely related forms of diabetes. We have now exploited genetic epistasis to examine how Hnf1α and Hnf4α functionally interact in pancreatic islets. Expression profiling in islets from either *Hnf1a^+/−^* or pancreas-specific *Hnf4a* mutant mice showed that the two transcription factors regulate a strikingly similar set of genes. We integrated expression and genomic binding studies and show that the shared transcriptional phenotype of these two mutant models is linked to common direct targets, rather than to known effects of Hnf1α on *Hnf4a* gene transcription. Epistasis analysis with transcriptomes of single- and double-mutant islets revealed that Hnf1α and Hnf4α regulate common targets synergistically. Hnf1α binding in *Hnf4a*-deficient islets was decreased in selected targets, but remained unaltered in others, thus suggesting that the mechanisms for synergistic regulation are gene-specific. These findings provide an *in vivo* strategy to study combinatorial gene regulation and reveal how Hnf1α and Hnf4α control a common islet-cell regulatory program that is defective in human monogenic diabetes.

## Author Summary

The transcriptional activity of each gene is typically determined by multiple transcription factors. This concept has been well established in studies of single genes. However, transcription factors do not simply regulate single genes, they also control broad gene programs that underlie cellular function and disease. Understanding how combinations of transcription factors interact at the level of cellular regulatory programs remains a challenge. Humans with mutations in the genes encoding for the transcription factors Hnf1α and Hnf4α develop similar forms of diabetes that result from abnormal insulin secretion, suggesting that the two factors might have related functions in insulin-producing islet-cells. We now show that Hnf1α or Hnf4α bind to a common set of genes and that islet-cells from mice in which either Hnf1α or Hnf4α has been selectively disrupted show abnormal expression of similar genes. By comparing the gene expression defects of mice with mutations in either *Hnf1a*, *Hnf4a*, or both genes, we determined that Hnf1α and Hnf4α regulate common target genes through synergistic mechanisms. These results thus provide insight into a regulatory network that fails in human diabetes. Similar genetic strategies can also be employed to unravel how other transcription factors interact functionally in native cellular contexts.

## Introduction

In all eukaryotic organisms a limited number of DNA binding transcriptional regulators determine a much greater number of genetic programs. This is made possible by a code whereby unique combinations of regulators define cellular fates or functions. The combinatorial nature of transcriptional regulation has been demonstrated in countless studies that have dissected individual gene regulatory regions [1]–[4]. A major underlying principle is that DNA-binding transcriptional activators often function synergistically due to cooperativity in binding or recruitment of regulatory complexes [1], [3]. Other common functional interactions include redundancy or antagonism between different factors binding to the same regulatory region [1]–[4].

A true understanding of transcriptional programs will require the dissection of transcription factor interactions in global cellular contexts, rather than in single genes. In recent years, the function of several mammalian transcription factors has been examined by profiling gene expression in genetically perturbed cells [5]. Such studies provide a broad inventory of genes that are dependent on selected transcription factors, but they do not in themselves reveal how different factors interact functionally. Other studies have determined the genomic binding sites of single or multiple transcription factors [6], [7]. However, knowing that a regulator binds to a gene does not clarify if the binding event leads to positive, negative, or no regulation. Numerous studies, in fact, suggest that a major fraction of transcription factor binding events might be functionally dispensable [8]–[13]. Similarly, when more than one factor binds to the same gene, several functional interactions are possible. New approaches are therefore necessary to understand how transcriptional regulators engage in the combinatorial interactions that regulate cellular programs.

The genetics of human diabetes provides a paradigm to study transcriptional programs in pancreatic β-cells [14]–[17]. Heterozygous mutations in several genes encoding DNA binding transcription factors cause autosomal dominant diabetes, or Maturity Onset Diabetes of the Young (MODY) [14]–[16], [18]. Mutations in *HNF1A* and *HNF4A* (encoding for hepatocyte nuclear factor 1α and 4α) are responsible for the most common form of monogenic diabetes [14], [15]. Despite transient differences in newborns, the diabetic phenotype in *HNF1A* and *HNF4A* patients shares many features, including similar disease progression curves, insulin secretory responses, and sensitivity to hypoglycemic drugs [18]. Human genetics therefore suggests that *HNF1A* and *HNF4A* may be involved in a common regulatory network in β-cells.

One simple explanation for the shared *HNF1A* and *HNF4A*-deficient phenotype is that Hnf1α regulates the transcription of the *Hnf4a* pancreas-specific promoter[19]–[21]. However, several lines of evidence point to additional regulatory interactions. For example, a large-scale binding study found that many Hnf1α-bound genes are also bound by Hnf4α [6]. Other studies have shown that Hnf1α physically interacts *in vitro* and *in vivo* with the Hnf4α AF2 domain [22]–[24]. Such interactions have been linked to observations that overexpression of Hnf1α inhibits Hnf4α-regulation of targets, and overexpression of Hnf4α inhibits Hnf1α function [23]–[25]. Other studies showed that Hnf4α can increase Hnf1α function in synthetic promoters that only contain an Hnf1α binding site [22], or in promoters containing binding sites for both factors [2], [26], [27]. Because so far most functional studies have employed overexpression systems in cultured non-β cell lines, the true functional consequences of Hnf1α/Hnf4α interactions in islet-cells remain unclear.

We have now developed a strategy to study the integrated function of Hnf1α and Hnf4α in pancreatic islet cells. We profiled gene expression in genetic models with weak phenotypes and show that Hnf1α and Hnf4α regulate a remarkably similar set of genes. Using binding studies and epistasis analysis of transcriptome phenotypes, we demonstrate that the common function of Hnf1α and Hnf4α in pancreatic islet cells is in part due to global synergistic interactions between the two factors at common direct targets. The results provide an approach to decipher transcriptional networks in mammalian cells, and reveal novel insight into a common regulatory program that underlies human monogenic diabetes.

## Results

### *Hnf4a*-deficient pancreatic islets exhibit an impaired transcriptional program

**Figure 1:**
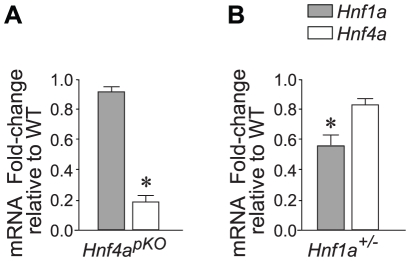
*Hnf1a* and *Hnf4a* expression in mutant models. (A,B) Expression of *Hnf1a* and *Hnf4a* mRNA in islets from (A) *Hnf4a^pKO^* and (B) *Hnf1a^+/−^* male mice. Results were normalized to *Hprt* mRNA and are expressed relative to littermate wild-type controls. * *P<*0.05.

The goal of this study was to understand the integrated transcriptional function of Hnf1α and Hnf4α in pancreatic islets. To study Hnf4α function, we generated pancreas-specific Hnf4a knock-out mice (Hnf4a^pKO^), and confirmed that this targeted deletion caused a marked reduction of Hnf4a gene mRNA in islet-cells (Figure 1A).

In keeping with previous studies of mice with β-cell specific ablation of *Hnf4a* (*Hnf4a* ^betaKO^) [28]–[30], *Hnf4a^pKO^* mice developed a mild complex phenotype, with very subtle glucose intolerance and a slightly reduced fasting glycemia (Figure S1).

Despite this mild metabolic phenotype, *Hnf4a^pKO^* mice showed a clear islet transcriptional phenotype (Table S1). Downregulated genes encoded for varied cellular roles, including the metabolism of steroids, glucose, and amino acids (Table S1 and Table S2). Others encoded for regulators of signal transduction and cell growth, consistent with a previous report in *Hnf4a^betaKO^* islets [31], or were in keeping with the proposed role of Hnf4α in epithelial differentiation [32] (Table S1 and Table S2). Upregulated genes included genes known to form part of the epithelial mesenchymal transition process (Table S2). Overall, the functional classes that were perturbed in *Hnf4a*-deficient islets were remarkably similar to those reported in *Hnf1a* ^-/-^ islets[11].

### *Hnf1a* haploinsufficient mice reveal *Hnf1a-*dependent transcription in islets

To study Hnf1α transcriptional function, we used *Hnf1a^+/−^* mice. As opposed to mice with homozygous *Hnf1a* mutations, which develop diabetes, this model has no documented *in vitro* or *in vivo* metabolic disturbances (Figure S2)[33]–[36]. Also in keeping with previous studies, *Hnf1a^+/−^* islets exhibit only marginal downregulation of Hnf4α (∼70–90% of normal values) (Figure 1B)[36]. *Hnf1a^+/−^* mice thus lack two elements that are thought to exert an indirect impact on islet gene expression in homozygous *Hnf1a* mutant islets. Array analysis revealed a transcriptional phenotype in *Hnf1a^+/−^* islets, with 196 non-redundant genes downregulated >1.5-fold in *Hnf1a^+/−^* islets at a nominal P value<0.01. We validated this dataset with gene-specific assays in 20 genes from independent *Hnf1a^+/−^* mice (Figure S4A). Furthermore, genes that were bound by Hnf1α and downregulated in homozygous *Hnf1a* mutant islets were significantly downregulated in *Hnf1a^+/−^* islets (Figure S4B). Thus, expression profiling in *Hnf1a^+/−^* islets provides a tool to assess the transcriptional function of Hnf1α in this tissue.

### *Hnf4a*- and *Hnf1a*-deficient islets share a common transcriptional signature

**Figure 2:**
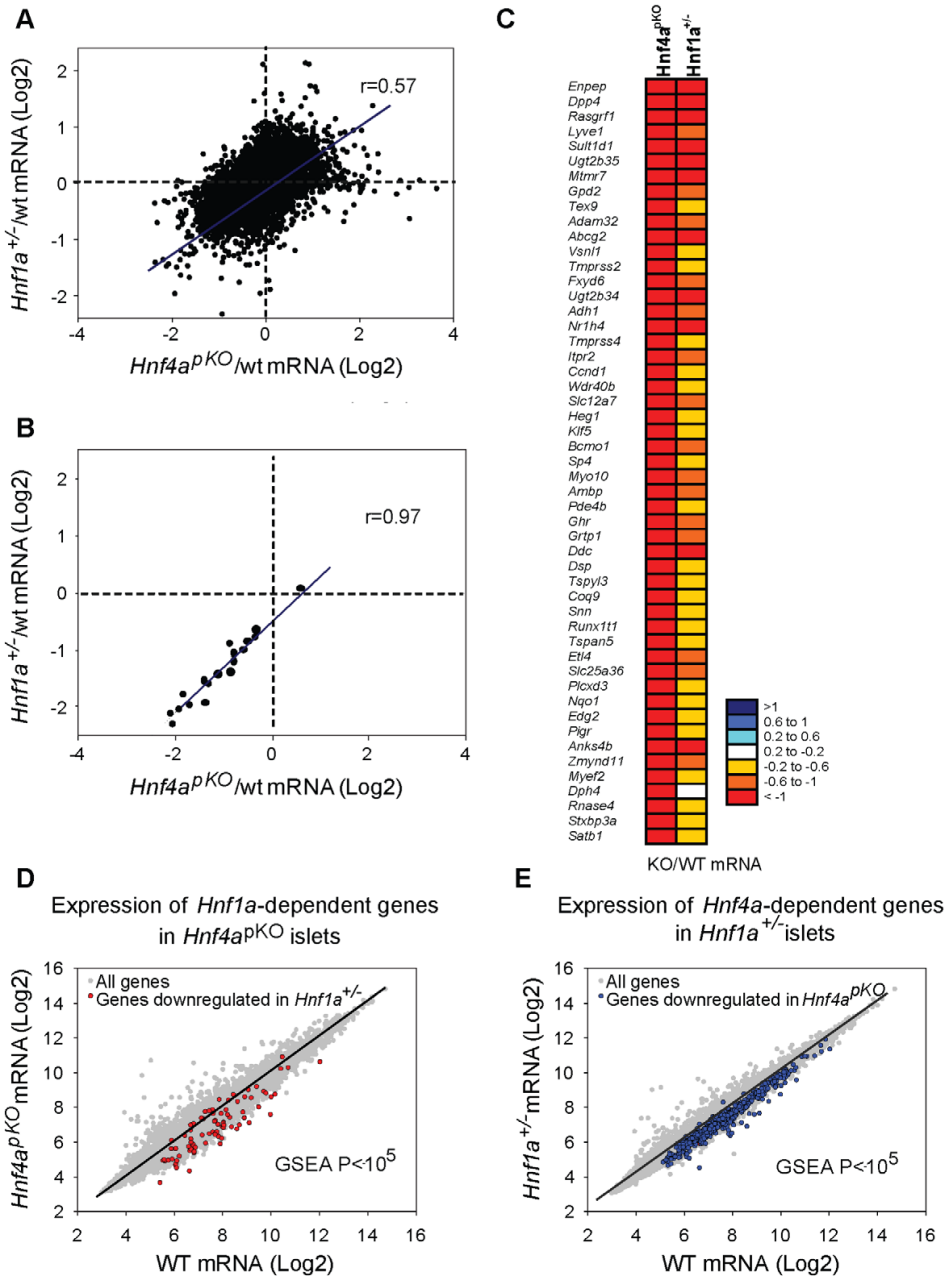
Hnf1α and Hnf4α regulate a common set of genes. (A) Correlation of mutant/wild-type Log2 gene expression ratios in *Hnf4a^pKO^* versus *Hnf1a^+/−^* islets. (B) Validation of 22 genes using gene-specific qPCR. (C) Heatmap of expression ratios in *Hnf4a^pKO^* and *Hnf1a^+/−^* islets for the 50 most downregulated genes in *Hnf4a^pKO^* islets. (D) Expression of *Hnf1a*-dependent genes in *Hnf4a^pKO^* islets. Grey dots represent average expression values of genes in *Hnf4a^pKO^* versus control islets. We superimposed red dots to show the subset of genes downregulated in *Hnf1a^+/−^* islets. (E) Expression of *Hnf4a*-dependent genes in *Hnf1a^+/−^* islets. Grey dots are expression values of all genes, superimposed blue dots are the subset of genes downregulated in *Hnf4a* ^pKO^ islets.

We next compared expression changes in *Hnf1a^+/−^* and *Hnf4a^pKO^* islets. This revealed a striking correlation between the two models (r = 0.57, P = 10^−6^) (Figure 2A–2C). Gene set enrichment analysis (GSEA) showed that genes that were significantly downregulated in *Hnf4a* ^pKO^ islets were downregulated in *Hnf1a^+/−^* islets (Figure 2D, P*<*0.001). Conversely, genes downregulated in *Hnf1a^+/−^* islets were downregulated in *Hnf4a^pKO^* islets (Figure 2E, P*<*0.001). Not surprisingly, gene expression changes were consistently lower in the *Hnf1a* haploinsufficient islets compared with islets with biallelic inactivation of *Hnf4a* (Figure 2A–2E). We confirmed the correlation with gene-specific assays (Figure 2B), and with an independent comparison of *Hnf1a^+/−^ versus Hnf4a^pKO^* mice of 16 rather than 8 weeks of age (not shown, and Table S1).

These common gene expression changes were unexpected, because *Hnf1a* expression in *Hnf4a^pKO^* islets was unperturbed (Figure 1A) (as previously shown for *Hnf4a^betaKO^* mice[28]–[30]) and *Hnf4a* expression was only marginally reduced in *Hnf1a^+/−^* islets (Figure 1B, and [36]). In conclusion, the analysis of models that minimize the impact of indirect perturbations showed that Hnf1α and Hnf4α regulate a common set of genes in pancreatic islets.

### Hnf1α targets are similarly impaired in *Hnf4a*- and *Hnf1a-*deficient islets

**Figure 3:**
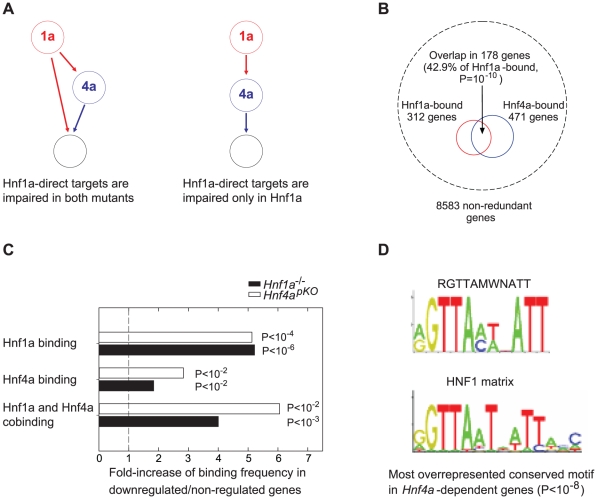
Expression of Hnf1α targets is impaired in *Hnf4a*-deficient islets. (A) Alternate models of Hnf1α and Hnf4α network structures that could potentially underlie the similar transcriptomes in *Hnf4a^pKO^* and *Hnf1a^+/−^* islets, and expected functional perturbation of Hnf1α bound genes in each case. (B) The analysis of previously reported [8], [11] mouse liver binding datasets showed that Hnf1α and Hnf4α preferentially bind the same genes, as reported in human islets and liver[6]. Hypergeometric distributions were tested to calculate significance values. (C) Hnf1α, Hnf4α and Hnf1α/Hnf4α binding were enriched in genes that were significantly downregulated 2-fold in *Hnf4a^pKO^* and *Hnf1a* ^-/-^ islets. Hypergeometric distributions were tested to calculate significance values. (D) Most significant over-represented evolutionary conserved sequence element in 10 Kb surrounding transcription start sites of genes that were downregulated in *Hnf4a^pKO^* islets. The canonical HNF1 matrix is shown below. Motifs matching Hnf4α, or Hnf1α and Hnf4α binding sequences were also overrepresented in genes downregulated in *Hnf4a^pKO^* and *Hnf1a* ^-/-^ islets, respectively (not shown).

Previous studies provide two possible mechanisms whereby *Hnf1a^+/−^ versus Hnf4a^pKO^* islets could exhibit a similar transcriptional phenotype (Figure 3A). One is that Hnf1α and Hnf4α frequently bind the same genes in human liver and islets [6]. We confirmed this finding using mouse liver binding datasets reported elsewhere [8], [11] (Figure 3B), after estimating that ∼75% of Hnf4α-bound genes in islets may also bound in liver (Figure. S8). However, co-occupancy does not *per se* explain the similar gene expression changes in the two mutant models, because for most genes bound by Hnf1α or Hnf4α, gene expression is not altered in the respective knock-out tissues [8], [11]. An alternate explanation for the similar transcriptional phenotypes is that Hnf1α regulates *Hnf4a* gene transcription in islets (Figure 3A) [11], [19]–[21]. In the current study we used heterozygous *Hnf1a* mutant islets because *Hnf4a* was not significantly altered (unlike homozygous *Hnf1a* mutants). However, it remained possible that a subtle decrease in *Hnf4a* expression in *Hnf1a^+/−^* islets caused the common transcriptional phenotype (which would thus result from a perturbation of Hnf4α in both models). We predicted that if this were true we should observe impaired expression of direct targets of Hnf4α in the two models, whereas the direct targets of the upstream factor of this hierarchy, Hnf1α, should be impaired only in *Hnf1a*-deficient islets (Figure 3A).

We thus tested if transcription of Hnf1α-bound genes was impaired in mice deficient for either factor. Hnf1α binding frequency was increased 5-fold among genes that were downregulated in homozygous *Hnf1a* mutant islets (Figure 3C). Remarkably, Hnf1α binding frequency was also increased 5-fold in promoters of genes that were downregulated in *Hnf4a*-deficient islets (Figure 3C). We also found that Hnf1α bound genes that were downregulated in *Hnf1a* ^-/-^ islets were downregulated in *Hnf4a*-deficient islets (Figure S5). *In silico* studies confirmed these findings, as the most overrepresented conserved motif in genes downregulated in *Hnf4a^pKO^* islets was identical to the canonical HNF1 binding sequence (Figure 3D). Thus, Hnf1α targets are impaired in *Hnf4a* deficient islets to a similar extent as in *Hnf1a* deficient islets.

Genes that were downregulated in both *Hnf4a*- and *Hnf1a-*deficient islets also showed significantly enriched Hnf4α binding and a 4 to 6-fold higher co-occupancy rate than non-regulated genes (Figure 3C). Collectively, these results argue that the shared transcriptome in our models is not due to the known *Hnf1a-Hnf4a* transcriptional hierarchy, and instead support that it is linked to the regulation of common target genes.

### Hnf1α and Hnf4α function is interdependent in pancreatic islets

**Figure 4:**
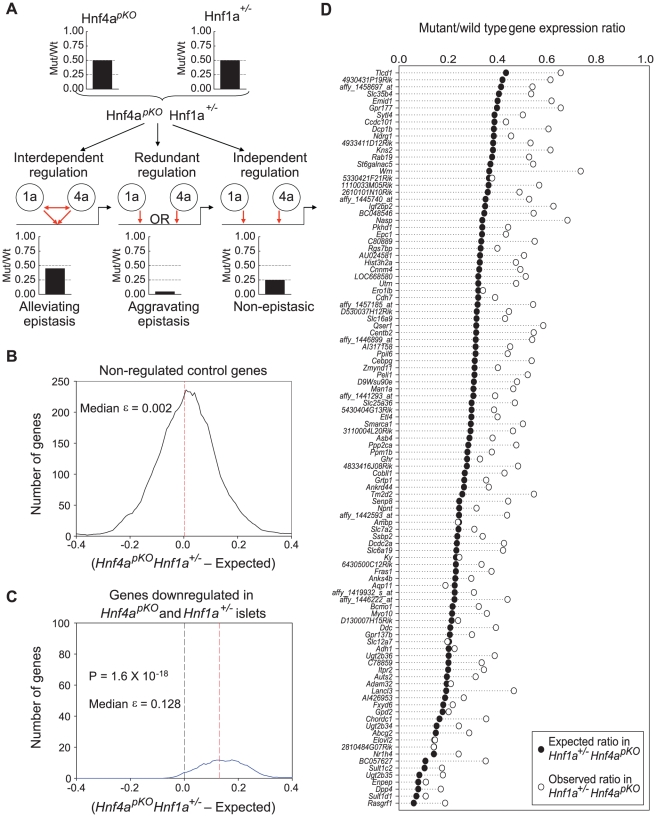
Epistasis reveals functional synergism between Hnf1α and Hnf4α. (A) Schematic representation of the genetic approach used to test functional interactions between *Hnf1a* and *Hnf4a* in a hypothetical gene that is downregulated 50% of wild type values in both *Hnf4a^pKO^* and *Hnf1a^+/−^* islets. (B,C) Distribution of ε values (see results for explanation) for control genes (B), or for all genes that were downregulated in both single mutant mice (C). (D) Observed gene expression ratios in *Hnf4a^pKO^ Hnf1a^+/−^* islets (white circles) and expected changes in a non-epistatic model (black circles) for each gene that was significantly downregulated in both single mutant mice.

The above findings were consistent with an interdependent function of Hnf1α and Hnf4α, or alternatively with the regulation of common genes in a mechanistically independent manner. To discriminate among these possibilities, we compared expression profiles in single mutant (*Hnf1a^+/−^* and *Hnf4a^pKO^*) *versus* double mutant (*Hnf1a^+/−^ Hnf4a^pKO^*) islets (Figure 4A). We predicted that if Hnf1α and Hnf4α act independently in any gene that is downregulated in both single mutant islets, the expression ratio in double mutant (*Hnf1a^+/−^ Hnf4a^pKO^*) islets should reflect the product of the two single mutant expression ratios. By contrast, if the two factors regulate a gene in an interdependent manner, the expression in double mutant islets should differ from this expectation (Figure 4A).

For each gene that was downregulated in both *Hnf1a^+/−^* and *Hnf4a^pKO^* single mutant islets we calculated an epistasis ε value that measures the deviation from expectation. An ε value > 0 indicates that the expression ratio in *Hnf1a^+/−^ Hnf4a^pKO^* islets is higher (less perturbed) than expected from the independent effects of the two single mutant values. As shown in Figure 4C, the distribution of ε values was unambiguously greater than 0. Figure 4D further illustrates this concept, showing that the perturbation of individual genes in *Hnf1a^+/−^ Hnf4a^pKO^* islets was epistatic (P = 10^−35^). Similar findings were confirmed in independent mice using qPCR (quantitative PCR) rather than oligonucleotide chips (Figure S6).

The high co-occupancy rate of Hnf1α and Hnf4α also raises the question whether these two factors might exert redundant functions at some targets. Because selecting genes that are downregulated in the single mutant islets can represent a bias against redundancy, we also performed this analysis in all genes that were downregulated >3 fold in double *Hnf1a^+/−^ Hnf4a^pKO^* mutant islets. Most such genes were markedly downregulated in the single mutants, and again had ε values exceeding 0 (Figure S7), thus showing that although Hnf1α and Hnf4α often bind to the same genes, their function is not redundant in islets. In keeping with these findings, the mild glucose intolerance phenotype observed in *Hnf4a^pKO^* mice was not further impaired in *Hnf1a^+/−^ Hnf4a^pKO^* mice (Figure S3). In summary, these results indicate that Hnf1α and Hnf4α bind to similar targets and act through interdependent regulatory mechanisms in islets, thus leading to a common transcriptional phenotype in *Hnf1a^+/−^* and *Hnf4a^pKO^* islets.

### Gene-specific mechanisms for interdependent activation

**Figure 5:**
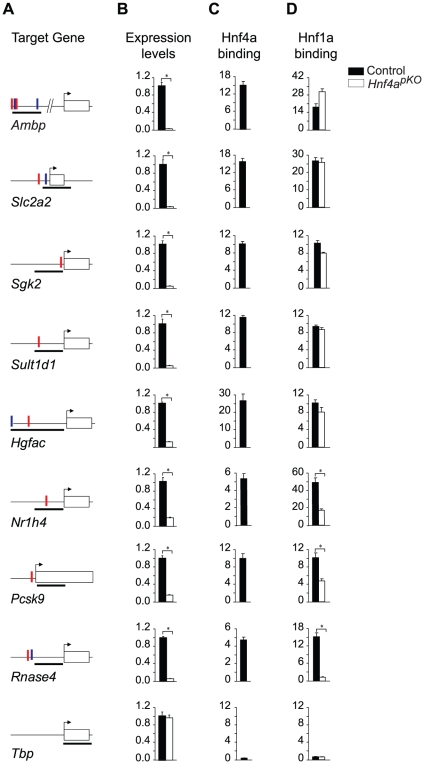
Gene-specific mechanisms for functional synergism. We tested Hnf1α binding in *Hnf4a*-deficient islets in 8 genes that are bound by Hnf1α and Hnf4α in wild type islets and are downregulated in *Hnf1a* and *Hnf4a*-deficient islets. Hnf1α binding in *Hnf4a*-deficient islets was unaltered in 5/8 genes examined, was partially reduced in two genes, and was abrogated in one gene. (A) Schematic representations of PCR products (black thick lines) used for Hnf1α and Hnf4α ChIPs, and high affinity HNF1 (red vertical lines) and HNF4 (blue vertical lines) binding sequences. (B) Gene expression in wild type and *Hnf4a*-deficient islets assayed by quantitative PCR. Results are normalized by expression levels of *Actb* mRNA, and are shown as fold-changes relative to wild type islets. (C,D) Hnf1α and Hnf4α binding in wild type (black bars) and *Hnf4a*-deficient (white bars) islets. Results are expressed as fold over *Actb* negative control regions. *Tbp* is shown as an independent negative control for both ChIP and gene expression studies. * *P<*0.05.

To assess the mechanisms underlying the interdependent function of Hnf1α and Hnf4α in common target genes, we examined whether their binding is interdependent. Because Hnf1α expression is unaltered in *Hnf4a*-deficient mouse islets, we were able to use this model to study whether Hnf4α is required for Hnf1α binding to common targets. We selected eight genes that we have previously shown are bound by Hnf1α and are functionally dependent on Hnf1α in islets [11](Figure 5A). All of them were also directly bound by Hnf4α in wild type islets (Figure 5C), and were markedly downregulated in *Hnf4a*-deficient islets (Figure 5B). Thus, all 8 selected genes were co-occupied and were functionally dependent on the two factors in islets. We next examined Hnf1α binding to these sites in *Hnf4a*-deficient islets. We found that in 5 of 8 genes, Hnf1α binding was maintained in islets that lack Hnf4α (Figure 5D). In two other *Hnf4a*-dependent genes, Hnf1α binding was significantly reduced in *Hnf4a*-deficient islets, and was completely abrogated in one case (Figure 5D). The difference between genes concerning Hnf1α binding in *Hnf4a*-deficient islets could not be linked to differences in the affinity of Hnf1α and Hnf4α binding sites. Thus, the synergistic activation of common targets by Hnf1α and Hnf4α can reflect gene-specific interdependent mechanisms at both binding and post-binding levels.

## Discussion

### Epistasis reveals functional interdependence of Hnf1α and Hnf4α in islets

We have addressed how transcription factors establish functional interactions in an *in vivo* context. To achieve this, we studied epistatic relationships of transcriptional phenotypes. Our approach follows recent studies that used epistasis of transcriptomes to study functional interactions between regulators of protein kinase A in Dictyostelium, Mediator subunits in yeast, and most recently to unravel yeast transcription factor networks [37]–[39]. Our use of epistasis is analogous to classic studies that studied synergism or redundancy between transcription factors by comparing cells transfected with reporter minigenes along with single versus multiple transcription factors[1]. In the reverse approach we employed, we studied the transcriptome of mice with single and double transcription factor mutations. This allowed us to study combinatorial function *in vivo*, in endogenous genes of primary mammalian cells. It also enabled a global analysis, rather than studying specific gene targets that do not necessarily reflect a predominant regulatory strategy. This approach complements studies that compare the genomic location of different transcription factors without assessing their functional interactions. Our analysis thus confirmed previous observations that Hnf1α and Hnf4α bind to common targets, and suggests that these two factors function as obligate interdependent regulators in pancreatic islets. We thus demonstrate a role for epistasis to unravel the function of transcription factor networks in mammalian cells.

### Value of weak genetic phenotypes to study regulatory networks

Previous studies showed that homozygous *Hnf1a* mutant mice exhibit full blown diabetes, in contrast to β-cell and pancreas-specific *Hnf4a* mutations which only result in glucose intolerance [21], [28], [29], [31], [34], [40]. In light of these differences it was somewhat unexpected that Hnf1α and Hnf4α regulate similar islet genes. We observed this coregulation using heterozygous *Hnf1a* and pancreas-specific *Hnf4a* mutations, which do not have common metabolic disturbances that could confound the comparison. They are also selective models: four studies have now shown that *Hnf4a*-deficient islets express normal Hnf1α levels (this study, and [28]–[30]), and, in contrast to *Hnf1a* ^-/-^ mice, Hnf4α levels are only minimally altered in *Hnf1a^+/−^* islets (this study, and [36]). Plausibly, differences in the phenotype of *Hnf1a* and *Hnf4a*-deficient models reported so far are due to the use of different types of genetic inactivation systems. More generally, we believe that weak genetic perturbations can be of great interest in studying transcription factor function, because although they only cause mild target expression changes, they are also less likely to disrupt downstream regulatory networks, thus limiting the magnitude of indirect effects.

### Hnf1α and Hnf4α regulate common targets

Most genes bound by Hnf4α or Hnf1α are not affected by mutations of these two factors[11], [19]. It was thus necessary to integrate binding studies with genetic perturbation models to understand the functional interactions between these transcriptional regulators.

**Figure 6:**
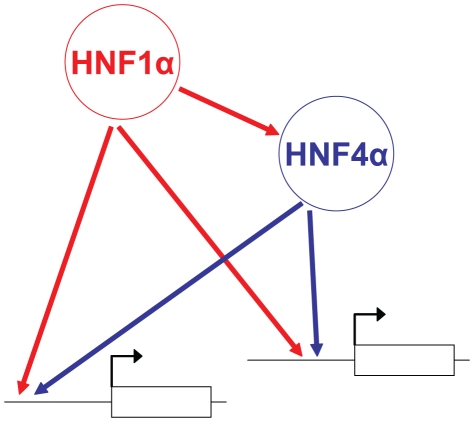
Model of the Hnf1α/Hnf4α regulatory network in pancreatic islets. In islets, Hnf1α controls *Hnf4a* gene transcription, while both Hnf1α and Hnf4α activate common targets synergistically.

We observed that functional Hnf1α targets were similarly perturbed in *Hnf4a*-deficient and *Hnf1a*-deficient islets. This suggests that Hnf4α regulates Hnf1α function, and discards that epistasis was simply due to the known transcriptional hierarchy in which *Hnf1a* is upstream of *Hnf4a*. Together with the high Hnf1α/Hnf4α co-occupancy rate reported here and previously in human liver and islets[6], these findings suggest that epistasis between *Hnf1a* and *Hnf4a* is at least in part due to interdependent interactions at common direct targets. We thus propose a model for the integrated function of Hnf1α and Hnf4α in islets whereby Hnf1α regulates *Hnf4a* transcription [19], [20], and furthermore both factors act as interdependent transcriptional partners in islet-cell targets (Figure 6). Importantly, this network model is not based solely on binding studies, but integrates information on the combinatorial functional interactions between these factors.

### Mechanisms underlying interdependent function

Several reports have demonstrated protein-protein interactions between Hnf1α and Hnf4α [22]–[24]. Such interactions can lead to functional inhibition [23], [24]. We show that although inhibitory consequences may be prevalent in other tissues or may occur in a small subset of genes, Hnf1α and Hnf4α largely activate genes synergistically in pancreatic islets. Earlier gene-specific studies have shown cooperative binding of Hnf1α and Hnf4α [2], [26], [27], while Hnf4α has been shown to co-activate a gene that is only directly bound by Hnf1α[22]. Our *in vivo* data showed that binding was interdependent in a subset of targets, but also showed that in many targets *Hnf4a*-deficiency does not entail decreased binding of Hnf1α. In the latter genes Hnf4α is likely required for post-binding functions of Hnf1α, such as the recruitment of co-regulatory complexes required for chromatin remodelling and/or assembly of the preinitiation complex. The mechanisms underlying functional synergism between Hnf1α and Hnf4α therefore appear to vary across target genes.

### Implications for monogenic diabetes

Our study predicts a common islet transcriptome defect in the pathophysiology of *HNF1A* and *HNF4A* diabetes. This is consistent with the clinically indistinguishable diabetic phenotype of adult *HNF1A* and *HNF4A* patients[18], [41]. An exception to this notion is that *HNF4A* mutations cause transient *in utero* and neonatal hyperinsulinism, which later evolves to decreased insulin secretion, whereas *HNF1A* mutations develop the latter phenotype without early hyperinsulinism [30]. This may result if Hnf4α has Hnf1α-independent functions during prenatal and neonatal developmental stages.

The interdependent function of Hnf1α and Hnf4α is also relevant to our understanding of how haploinsufficiency of *HNF1A* and *HNF4A* leads to β-cell dysfunction and diabetes. We previously proposed that *HNF1A* or *HNF4A* haploinsufficiency could lead to the disruption of a Hnf1α/Hnf4α positive cross-regulatory network in β-cells[42]. Our current model depicted in Figure 6 provides new elements to assess the consequences of haploinsufficiency for the complex Hnf1α/Hnf4α network. Synergistic Hnf1α/Hnf4α-dependent activation is expected to result in steeper activator-response curves, and thus greater vulnerability to decreased gene dosage. This may be more pronounced in islet-cells, where Hnf1α and Hnf4α concentrations are much lower than in liver and other tissues that are not clinically afflicted in MODY[11], [43]. Haploinsufficiency of either *HNF1A* or *HNF4A* may in this manner disrupt the function of both HNF1α and HNF4α and the common transcriptional program, which include essential genes for the proper function of pancreatic islets. In conclusion, these studies provide an approach to understand the *in vivo* function of a regulatory network, and increase our understanding of the mechanisms underlying monogenic diabetes.

## Materials and Methods

### Mouse models and isolation of cells

*Hnf4a* ^LoxP^ mice were obtained from The Jackson Laboratory[44], *Hnf1a^+/−^* mice were provided by Frank Gonzalez (NCI)[34]. *Pdx1* ^Cre^ transgenic mice were provided by Pedro Herrera (U. Geneva) [45]. All studies were performed according to procedures approved by the institutional animal care and use committee. Animals were maintained on C57B/l6 backgrounds and genotyped as described [40]. For *Hnf4a* studies, *Hnf4a* ^LoxP^ littermates lacking *Pdx1* ^Cre^ and *Pdx1* ^Cre^ mice lacking *Hnf4a* ^LoxP^ alleles were used as controls unless stated otherwise. Pancreatic islets from 2- to 4-month old mice were isolated as described [40]. Islets were cultured for 48 hr at 37°C, 5% CO2 in RPMI (Invitrogen) containing 11mM glucose supplemented with 10% FCS.

### Glucose tolerance

Animals were fasted overnight and injected glucose intraperitoneally (2 gm/Kg). Glucose was measured from the tail vein at 0,15,30,60 and 120 min. Fasted plasma insulin was measured by ELISA (Mercodia).

### Gene expression analysis

RNA from purified islets was isolated with Trizol (Invitrogen) and tested with an Agilent 2100 Bioanalyzer to ascertain RNA integrity. The reduction of *Hnf4a* mRNA in *Hnf4a* ^pKO^ mice varied between 5–50% of wild type islets, most likely due the inherent variability of Cre-based recombination. We thus assessed *Hnf4a* mRNA by semiquantitative PCR [46] and used samples with >80% reduction for further analysis.

For each array replicate, RNAs from 2–4 male mice were pooled, and 50 ng was used in two cycles of cDNA synthesis for hybridization of Affymetrix 430 2.0 arrays. For epistasis experiments we used 8 week-old male mice. We separately compared 16 week-old male *Hnf4a* ^LoxP^ and control mice. Three arrays (a total of 8–12 mice) were analyzed per genotype, normalized with RMA, and analyzed with the LIMMA package to identify downregulated genes using a multiple test adjusted P value <0.05. To select genes downregulated in *Hnf1a^+/−^* islets we used a nominal (unadjusted) *P<*0.01 threshold, and validated this set with gene-specific assays. Gene-specific expression was assessed either with Taqman Low Density Arrays (Applied Biosystems) using unpooled islet RNA samples from 2–3 additional mice per genotype, or by qPCR using SybrGreen detection system as described [11]. Gene expression datasets are available in ArrayExpress (http://www.ebi.ac.uk) (Accession number: E-MEXP-1729).

### Chromatin immunoprecipitation (ChIP) assays

Approximately 2000 purified islets from *Hnf4a* ^LoxP^ *Pdx1* ^Cre^ and littermates lacking the *Pdx1* ^Cre^ transgene were used for ChIP assays essentially as described [11], [19]. For each genotype we processed islets from two independent pools of ∼10 mice separately, we measured in duplicate the enrichment of immunoprecipitated DNA relative to input DNA, and corrected for the same values obtained in *Actb* as a negative control gene.

### Computational sequence analysis

We used oPOSSUM [47], which computes a Fisher exact test to measure over-representation of sequence elements in a gene set relative to a background comprising all genes. We analyzed evolutionary conserved sequences 5 Kb upstream and 5 Kb downstream of transcription start sites of all downregulated genes (M<−0.6), and searched for conserved sequence elements as described in [48]. The empirical recommendations to identify binding sites oPOSSUM are a Z-score>10 and a Fisher P value<0.01. Overrepresented motifs were tested against the JASPAR CORE database of binding site profiles.

### Epistasis and statistical analyses

Our analysis of epistasis of transcriptome phenotypes is based on previous large-scale studies of epistasis among yeast mutants regulating cell growth [49], [50]. We selected 105 genes that were downregulated (M<−0.6) in both *Hnf1a^+/−^* and *Hnf4a* ^pKO^ islets, and compared gene expression changes with that of double mutant islets. For each gene we calculated an ε epistasis value that measures the deviation of the *observed* mutant/wild type expression ratio (R) in *Hnf1a^+/−^ Hnf4a^pKO^* double mutant islets from the *expected* ratio based on the product of the two single mutant values (ε = R *Hnf1a^+/−^ Hnf4a^pKO^* - [R *Hnf1a^+/−^* x R *Hnf4a^pKO^*]). We performed a similar analysis for a control set of genes that showed no regulation in the two single mutant islets (M-1.1 to 1.1, p>0.2,). We calculated statistical significance with Student's t test, comparing the experimental and control ε value distributions, or with a paired Student's t test, comparing *Hnf1a^+/−^ Hnf4a^pKO^* R values to expected [R *Hnf1a^+/−^* x R *Hnf4a^pKO^*] values in each gene.

Enrichment of functional annotations was examined with GSEAv2.0 (http://www.broad.mit.edu/gsea/) using gene sets as the permutation type and 1000 permutations, and with DAVID (http://david.abcc.ncifcrf.gov/). Statistical significance in binding comparisons was calculated with two-sided Fisher's exact test, or by testing the Hypergeometric distribution.

## Supporting Information

Figure S1 Pancreatic *Hnf4a*-deficiency causes a mild alteration of glucose tolerance and fasting glycemia. (A,B) Fasting blood glucose and insulin in 8 week-old male wild type (black) *versus Hnf4a* ^pKO^ (white) littermate mice. (C) Intraperitoneal glucose tolerance test in wild type *versus Hnf4a* ^pKO^ male mice at 8 weeks of age. All experiments were performed after an overnight fast. Values are mean ± SEM. * Student's test P<0.05; n = 8-15 animals per group in each experiment.
(0.18 MB PDF)

Figure S2 *Hnf1a* haploinsufficiency does not alter glucose tolerance. (A,B) Fasting blood glucose and insulin in 52 week-old male wild type (white) versus *Hnf1a* ^+/-^ (grey) littermate mice. (C) Intraperitoneal glucose tolerance test in wild type versus *Hnf1a* ^+/-^ male mice at 52 weeks of age. All experiments were performed after an overnight fast. Values are mean ± SEM. n = 10-25 animals per group in each experiment. No changes in glucose tolerance were observed at 8-48 weeks of age (not shown).
(0.16 MB PDF)

Figure S3 Glucose homeostasis is similar in *Hnf4a* ^pKO^ and double mutant mice. (A) Fasting blood glucose in 10 week-old male wild type (black), *Hnf1a* ^+/-^ (grey), *Hnf4a* ^pKO^ (white) versus *Hnf1a* ^+/-^;*Hnf4a* ^pKO^ (grey with white dots) littermate mice. (B) Blood glucose levels 30 minutes after intraperitoneal glucose administration. The results show that consistent with the epistatic interactions revealed in transcriptome comparisons, the glucose homeostasis abnormalities do not differ in double mutant and *Hnf4a*-deficient islets. Values are mean ± SEM. Each group is formed by 8-12 male mice. *P<0.05 relative to wild type mice.
(0.17 MB PDF)

Figure S4 (A) Gene-specific qPCR (Taqman) analysis of 20 genes that showed downregulation in *Hnf1a* ^+/-^ islets using Affymetrix gene chips at a nominal significance value of P<0.01. This result confirms that this statistical threshold selects genes that are downregulated in *Hnf1a* ^+/-^ islets. (B) Assessment of Affymetrix gene expression ratios in *Hnf1a* ^+/-^ islets for Hnf1α-bound genes that are downregulated >2-fold in *Hnf1a* ^-/-^ islets [11]. This result shows that the direct essential functions of Hnf1α in islets are captured by expression profiling of *Hnf1a* ^+/-^ islets.
(0.14 MB PDF)

Figure S5 Expression in *Hnf4a* ^pKO^ islets for the set of genes comprised of Hnf1α-bound genes that are downregulated >2-fold in *Hnf1a* ^-/-^ islets. Grey dots represent average expression values of all genes in *Hnf4a^pKO^* versus control islets. Red dots are values for Hnf1α-bound genes downregulated in *Hnf1a* ^-/-^ islets.
(2.12 MB PDF)

Figure S6 Epistasis analysis validation in *Hnf1a*- and *Hnf4a*-dependent genes. We selected a random set of genes that were downregulated by >25% in both single *Hnf1a^+/-^* and *Hnf4a* ^pKO^ mutant islets in Affymetrix experiments. We used gene-specific qPCR to measure expression in the four experimental genotypes, and compared the observed and expected expression ratios in *Hnf4a* ^pKO^ *Hnf1a* ^+/-^ islets. The results represent the average of 2-3 non-pooled samples per genotype, all of which differed from the samples used in Affymetrix chip analysis. P value was obtained by a Paired Student's t test.
(0.23 MB PDF)

Figure S7 Lack of evidence for functional redundancy between Hnf1α and Hnf4α. We studied all genes that were downregulated >3 fold in *Hnf4a* ^pKO^ *Hnf1a* ^+/-^ islets to avoid a bias against redundancy due to the selection of genes that were downregulated in single mutant islets. This set of genes (A) was almost invariably also downregulated in the single mutant islets, and (B) showed in most cases epsilon values >0, thus discarding significant redundant functions between Hnf1α and Hnf4α in islets.
(0.14 MB PDF)

Figure S8 ChiP-chip analysis of Hnf4α in pancreatic islets. Representation of genes bound at Log2 M>0.6 in a technical replicate BCBC promoter microarray hybridization experiment using pancreatic islets, and the corresponding binding ratios in hepatocytes. Experiments were performed essentially as in [8]. The results show that 76% of genes bound in islets showed concordant binding at M>0.6 in liver.
(0.14 MB PDF)

Table S1 Summary of gene expression findings for genes downregulated >2 fold in *Hnf4a* ^pKO^ islets.
(0.08 MB PDF)

Table S2 Overrepresentation of functional classes among regulated genes in *Hnf4a* ^pKO^ and *Hnf1a* ^+/-^ islets.
(0.03 MB PDF)
