# Supplementary figures and images for: Epistasis of Transcriptomes Reveals Synergism between Transcriptional Activators Hnf1α and Hnf4α
Source: PLoS Genet. 2010 May 27;6(5):e1000970. doi: 10.1371/journal.pgen.1000970 (PMC2877749; doi:10.1371/journal.pgen.1000970)

**A**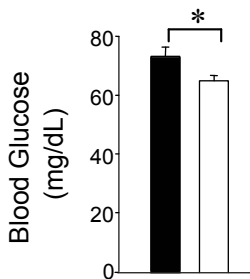**B**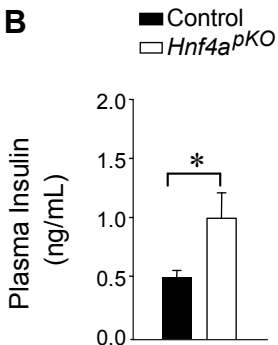**C**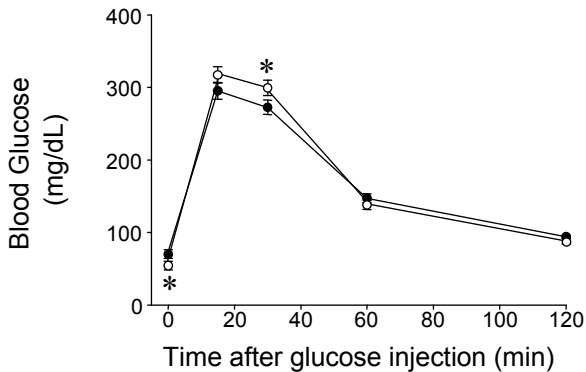

Supplement: Figure S1 — Pancreatic Hnf4a-deficiency causes a mild alteration of glucose tolerance and fasting glycemia. (A,B) Fasting blood glucose and insulin in 8 week-old male wild type (black) versus Hnf4a pKO (white) littermate mice. (C) Intraperitoneal glucose tolerance test in wild type versus Hnf4a pKO male mice at 8 weeks of age. All experiments were performed after an overnight fast. Values are mean ± SEM. * Student's test P<0.05; n = 8-15 animals per group in each experiment. (0.18 MB PDF) [file pgen.1000970.s001.pdf]

**A**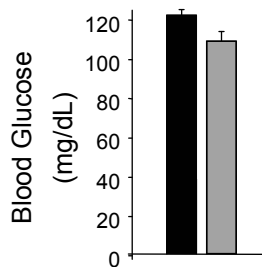**B**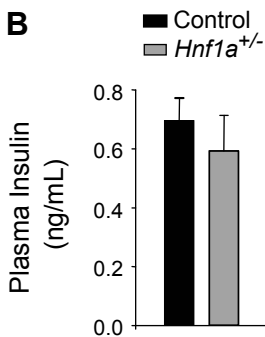**C**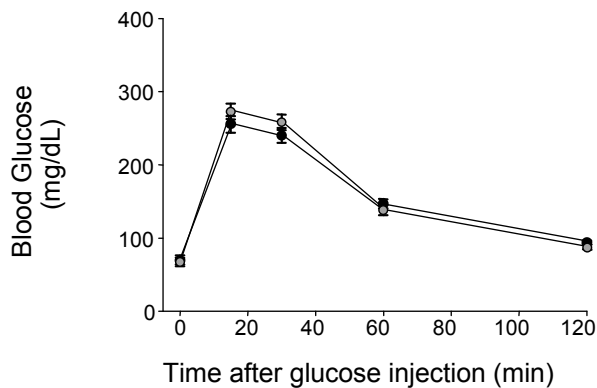

Supplement: Figure S2 — Hnf1a haploinsufficiency does not alter glucose tolerance. (A,B) Fasting blood glucose and insulin in 52 week-old male wild type (white) versus Hnf1a +/- (grey) littermate mice. (C) Intraperitoneal glucose tolerance test in wild type versus Hnf1a +/- male mice at 52 weeks of age. All experiments were performed after an overnight fast. Values are mean ± SEM. n = 10-25 animals per group in each experiment. No changes in glucose tolerance were observed at 8-48 weeks of age (not shown). (0.16 MB PDF) [file pgen.1000970.s002.pdf]

**A**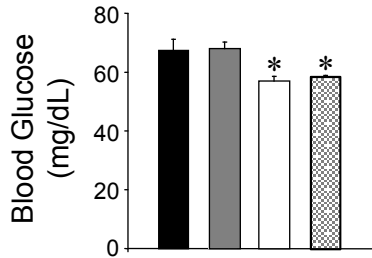**B**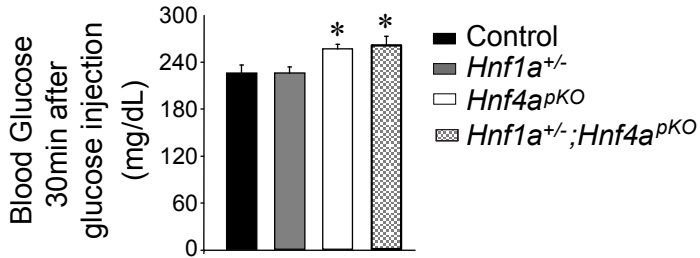

Supplement: Figure S3 — Glucose homeostasis is similar in Hnf4a pKO and double mutant mice. (A) Fasting blood glucose in 10 week-old male wild type (black), Hnf1a +/- (grey), Hnf4a pKO (white) versus Hnf1a +/-;Hnf4a pKO (grey with white dots) littermate mice. (B) Blood glucose levels 30 minutes after intraperitoneal glucose administration. The results show that consistent with the epistatic interactions revealed in transcriptome comparisons, the glucose homeostasis abnormalities do not differ in double mutant and Hnf4a-deficient islets. Values are mean ± SEM. Each group is formed by 8-12 male mice. *P<0.05 relative to wild type mice. (0.17 MB PDF) [file pgen.1000970.s003.pdf]

**A**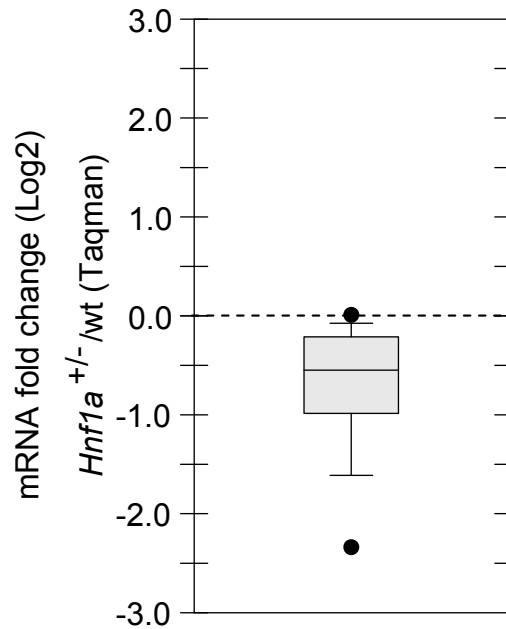**B**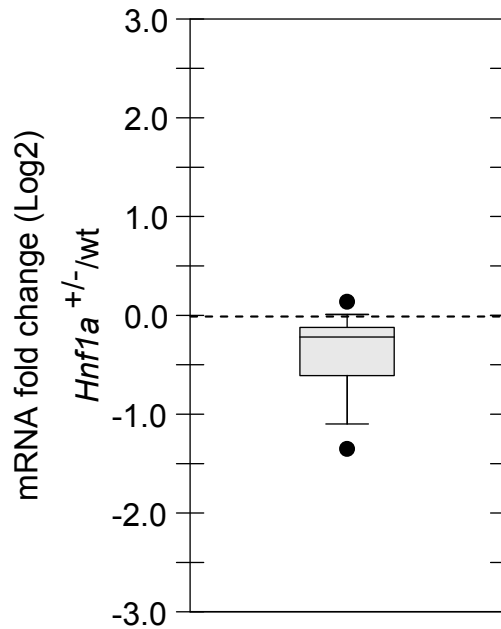

Supplement: Figure S4 — (A) Gene-specific qPCR (Taqman) analysis of 20 genes that showed downregulation in Hnf1a +/- islets using Affymetrix gene chips at a nominal significance value of P<0.01. This result confirms that this statistical threshold selects genes that are downregulated in Hnf1a +/- islets. (B) Assessment of Affymetrix gene expression ratios in Hnf1a +/- islets for Hnf1α-bound genes that are downregulated >2-fold in Hnf1a -/- islets [11]. This result shows that the direct essential functions of Hnf1α in islets are captured by expression profiling of Hnf1a +/- islets. (0.14 MB PDF) [file pgen.1000970.s004.pdf]

# Expression of Hnf1 $\alpha$ -bound *Hnf1a*-dependent genes in *Hnf4a*<sup>pKO</sup> islets

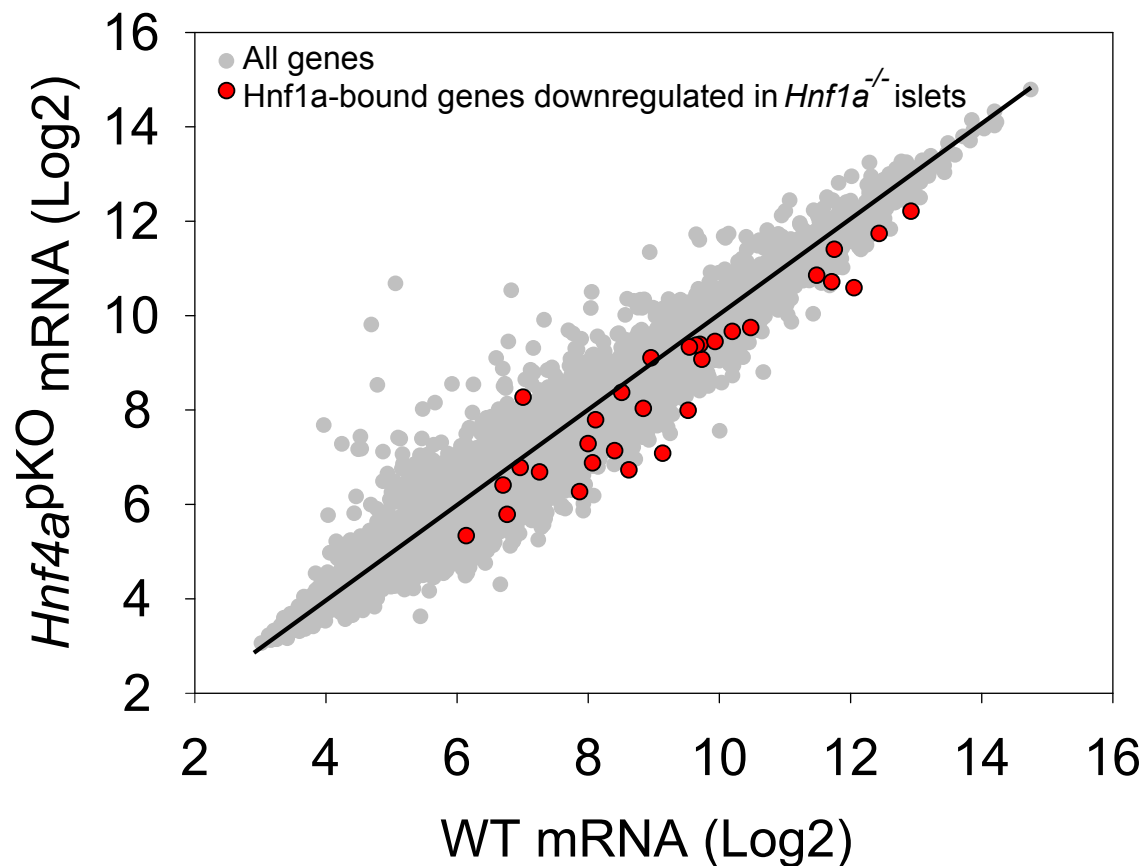

Supplement: Figure S5 — Expression in Hnf4a pKO islets for the set of genes comprised of Hnf1α-bound genes that are downregulated >2-fold in Hnf1a -/- islets. Grey dots represent average expression values of all genes in Hnf4apKO versus control islets. Red dots are values for Hnf1α-bound genes downregulated in Hnf1a -/- islets. (2.12 MB PDF) [file pgen.1000970.s005.pdf]

# Mutant/wild type

## gene expression ratio (Taqman)

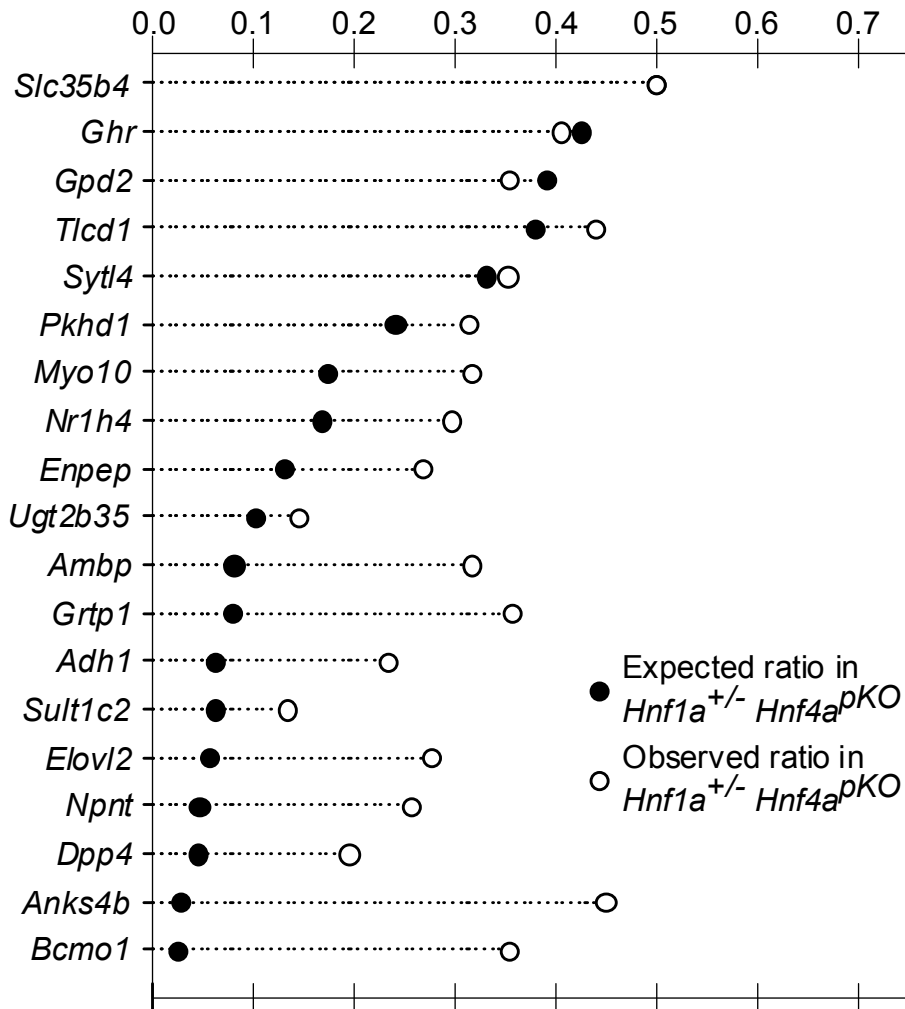

Supplement: Figure S6 — Epistasis analysis validation in Hnf1a- and Hnf4a-dependent genes. We selected a random set of genes that were downregulated by >25% in both single Hnf1a+/- and Hnf4a pKO mutant islets in Affymetrix experiments. We used gene-specific qPCR to measure expression in the four experimental genotypes, and compared the observed and expected expression ratios in Hnf4a pKO Hnf1a +/- islets. The results represent the average of 2-3 non-pooled samples per genotype, all of which differed from the samples used in Affymetrix chip analysis. P value was obtained by a Paired Student's t test. (0.23 MB PDF) [file pgen.1000970.s006.pdf]

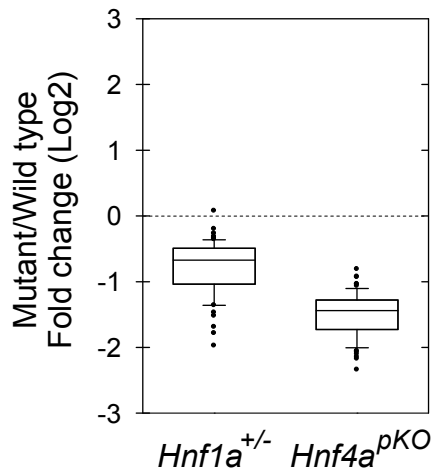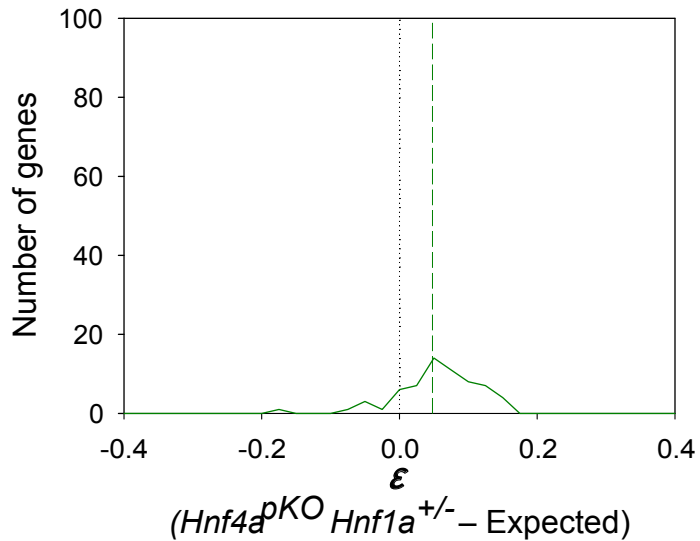

Supplement: Figure S7 — Lack of evidence for functional redundancy between Hnf1α and Hnf4α. We studied all genes that were downregulated >3 fold in Hnf4a pKO Hnf1a +/- islets to avoid a bias against redundancy due to the selection of genes that were downregulated in single mutant islets. This set of genes (A) was almost invariably also downregulated in the single mutant islets, and (B) showed in most cases epsilon values >0, thus discarding significant redundant functions between Hnf1α and Hnf4α in islets. (0.14 MB PDF) [file pgen.1000970.s007.pdf]

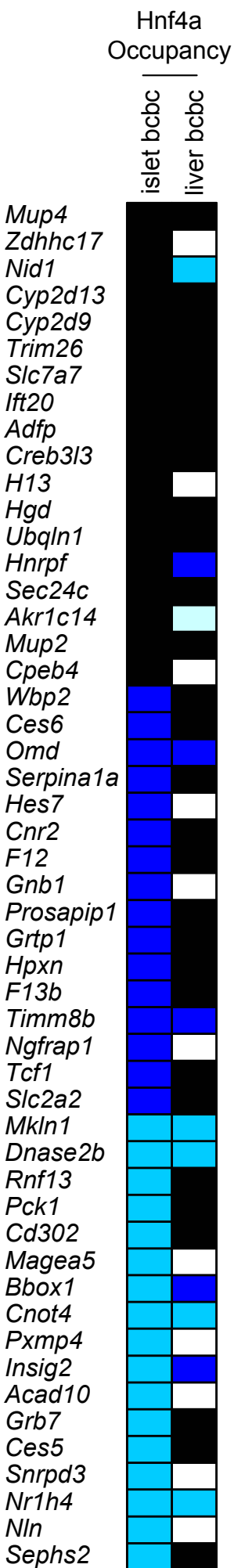

M value

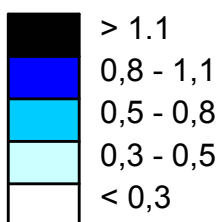

Supplement: Figure S8 — ChiP-chip analysis of Hnf4α in pancreatic islets. Representation of genes bound at Log2 M>0.6 in a technical replicate BCBC promoter microarray hybridization experiment using pancreatic islets, and the corresponding binding ratios in hepatocytes. Experiments were performed essentially as in [8]. The results show that 76% of genes bound in islets showed concordant binding at M>0.6 in liver. (0.14 MB PDF) [file pgen.1000970.s008.pdf]
