# Supplementary material for: Epistasis of Transcriptomes Reveals Synergism between Transcriptional Activators Hnf1α and Hnf4α
Source: PLoS Genet. 2010 May 27;6(5):e1000970. doi: 10.1371/journal.pgen.1000970 (PMC2877749; doi:10.1371/journal.pgen.1000970)
Supplement: Table S1 — Summary of gene expression findings for genes downregulated >2 fold in Hnf4a pKO islets. (0.08 MB PDF) [file pgen.1000970.s009.pdf]

**SUPPLEMENTARY TABLE 1** Summary expression findings for genes with known functions that are downregulated >2 fold in 8 wk old *Hnf4a*$^{pKO}$ islets

| | | | Gene regulation in mutant/control islets | | | | | |
|---|---|---|---|---|---|---|---|---|
| | | | ***Hnf4a*$^{pKO}$ islets (16 wk)** | | ***Hnf4a*$^{pKO}$ islets (8 wk)** | | ***Hnf1a*$^{+/-}$ islets (8 wk)** | |
| Probe Set ID | Gene Symbol | Gene Name | M (Log2) | p value | M (Log2) | p value | M (Log2) | p value |
| 1422906_at | *Abcg2* | ATP-binding cassette, sub-family G (WHITE), member 2 | -0.46 | 5.0E-02 | -1.72 | 1.1E-06 | -1.05 | 2.5E-04 |
| 1437376_at | *Adam32* | a disintegrin and metallopeptidase domain 32 | -1.96 | 5.9E-06 | -1.78 | 8.2E-05 | -0.61 | 2.5E-02 |
| 1416225_at | *Adh1* | alcohol dehydrogenase 1 (class I) | -1.58 | 3.7E-05 | -1.53 | 3.2E-04 | -0.80 | 8.2E-03 |
| 1416649_at | *Ambp* | alpha 1 microglobulin/bikunin | -1.19 | 2.1E-05 | -1.33 | 7.9E-04 | -0.73 | 1.1E-03 |
| 1434856_at | *Ankrd44* | ankyrin repeat domain 44 | -0.70 | 3.2E-04 | -1.28 | 4.9E-05 | -0.66 | 4.6E-03 |
| 1453042_at | *Anks4b* | ankyrin repeat and sterile alpha motif domain containing 4B | -1.18 | 1.3E-04 | -1.07 | 1.2E-03 | -1.08 | 2.7E-05 |
| 1447606_x_at | *Aqp11* | aquaporin 11 | -1.32 | 2.4E-04 | -1.45 | 1.3E-04 | -0.72 | 1.3E-03 |
| 1449907_at | *Bcmo1* | beta-carotene 15,15'-monooxygenase | -1.04 | 5.7E-04 | -1.38 | 2.3E-05 | -0.84 | 2.6E-04 |
| 1424547_at | *Car10* | carbonic anhydrase 10 | -0.83 | 2.6E-03 | -1.25 | 8.6E-04 | -0.18 | 1.0E-01 |
| 1448698_at | *Ccnd1* | cyclin D1 | -1.20 | 7.3E-05 | -1.45 | 8.1E-04 | -0.34 | 4.2E-02 |
| 1444438_at | *Cib3* | calcium and integrin binding family member 3 | -1.18 | 3.5E-06 | -1.16 | 1.9E-03 | -0.36 | 3.9E-01 |
| 1429166_s_at | *Clmn* | calmin | -0.90 | 7.3E-04 | -1.33 | 2.9E-05 | -0.50 | 4.9E-03 |
| 1434172_at | *Cnr1* | cannabinoid receptor 1 (brain) | -2.56 | 6.5E-06 | -1.86 | 3.2E-03 | -0.47 | 1.2E-01 |
| 1454795_at | *Cobll1* | Cobl-like 1 | -0.58 | 2.1E-03 | -1.31 | 1.4E-05 | -0.62 | 5.9E-03 |
| 1428134_at | *Coq9* | coenzyme Q9 homolog (yeast) | -0.95 | 1.6E-03 | -1.19 | 1.8E-05 | -0.23 | 1.6E-01 |
| 1459661_at | *Dcdc2a* | doublecortin domain containing 2a | -0.98 | 3.2E-06 | -1.21 | 6.1E-06 | -0.90 | 1.5E-04 |
| 1426215_at | *Ddc* | dopa decarboxylase | -0.50 | 7.5E-03 | -1.20 | 2.8E-03 | -1.09 | 2.6E-03 |
| 1424549_at | *Degs2* | degenerative spermatocyte homolog 2 (Drosophila), lipid desaturase | -1.26 | 2.1E-04 | -1.20 | 5.3E-05 | -0.24 | 3.4E-01 |
| 1451389_at | *Dnajc24* | DnaJ (Hsp40) homolog, subfamily C, member 24 | -0.69 | 1.4E-03 | -1.01 | 8.2E-06 | -0.20 | 1.0E-01 |
| 1416697_at | *Dpp4* | dipeptidylpeptidase 4 | -1.81 | 3.7E-07 | -2.18 | 8.8E-05 | -1.52 | 1.5E-04 |
| 1435493_at | *Dsp* | desmoplakin | -1.24 | 9.0E-06 | -1.20 | 1.5E-03 | -0.20 | 2.0E-01 |
| 1416444_at | *Elovl2* | elongation of very long chain fatty acids (FEN1/Elo2, SUR4/Elo3, yeast)-lik | -1.47 | 3.0E-05 | -1.62 | 2.6E-05 | -1.20 | 5.4E-03 |
| 1448649_at | *Enpep* | glutamyl aminopeptidase | -1.38 | 1.3E-03 | -2.34 | 3.2E-06 | -1.35 | 1.1E-04 |
| 1434582_at | *Erc2* | ELKS/RAB6-interacting/CAST family member 2 | -0.67 | 2.0E-04 | -1.01 | 2.0E-05 | -0.49 | 9.3E-03 |
| 1426880_at | *Etl4* | enhancer trap locus 4 | -0.77 | 1.5E-04 | -1.14 | 1.9E-04 | -0.65 | 1.7E-03 |
| 1452198_at | *Fbxl10* | F-box and leucine-rich repeat protein 10 | -1.45 | 5.4E-07 | -1.44 | 1.4E-05 | -0.56 | 2.0E-02 |
| 1439019_at | *Fras1* | Fraser syndrome 1 homolog (human) | -1.50 | 6.0E-05 | -1.40 | 3.8E-04 | -0.74 | 2.4E-03 |
| 1417343_at | *Fxyd6* | FXYD domain-containing ion transport regulator 6 | -1.96 | 2.9E-05 | -1.59 | 2.3E-03 | -0.90 | 1.5E-02 |
| 1451501_a_at | *Ghr* | growth hormone receptor | -0.71 | 5.5E-04 | -1.27 | 8.8E-04 | -0.61 | 5.2E-03 |
| 1428875_at | *Golim4* | golgi integral membrane protein 4 | -0.74 | 2.2E-04 | -1.20 | 2.0E-04 | -0.27 | 2.5E-01 |
| 1417434_at | *Gpd2* | glycerol phosphate dehydrogenase 2, mitochondrial | -1.60 | 6.7E-06 | -1.85 | 2.4E-06 | -0.67 | 1.1E-02 |
| 1437618_x_at | *Gpr85* | G protein-coupled receptor 85 | -0.69 | 6.4E-04 | -1.03 | 1.3E-04 | -0.33 | 5.9E-02 |
| 1439150_x_at | *Grtp1* | GH regulated TBC protein 1 | -1.14 | 2.1E-05 | -1.25 | 1.3E-03 | -0.68 | 3.6E-03 |
| 1439926_at | *Heg1* | HEG homolog 1 (zebrafish) | -0.61 | 2.9E-03 | -1.40 | 4.0E-05 | -0.51 | 1.2E-02 |
| 1424833_at | *Itpr2* | inositol 1,4,5-triphosphate receptor 2 | -0.57 | 2.8E-03 | -1.45 | 7.4E-05 | -0.90 | 1.1E-03 |
| 1451021_a_at | *Klf5* | Kruppel-like factor 5 | -0.62 | 1.6E-04 | -1.38 | 1.5E-04 | -0.55 | 4.6E-03 |
| 1440435_at | *Ky* | kyphoscoliosis peptidase | -1.79 | 1.7E-07 | -1.43 | 2.8E-04 | -0.70 | 4.7E-03 |
| 1437268_at | *Lancl3* | LanC lantibiotic synthetase component C-like 3 (bacterial) | -1.28 | 1.9E-04 | -1.35 | 2.0E-03 | -1.05 | 4.0E-04 |
| 1448606_at | *Lpar1* | lysophosphatidic acid receptor 1 | -0.53 | 3.4E-04 | -1.09 | 9.5E-04 | -0.40 | 1.9E-02 |
| 1429379_at | *Lyve1* | lymphatic vessel endothelial hyaluronan receptor 1 | -1.47 | 5.7E-04 | -2.07 | 1.4E-04 | -0.72 | 9.1E-02 |
| 1427025_at | *Mtmr7* | myotubularin related protein 7 | -0.54 | 4.7E-04 | -1.89 | 1.5E-04 | -1.96 | 8.3E-02 |
| 1425350_a_at | *Myef2* | myelin basic protein expression factor 2, repressor | -0.86 | 3.8E-04 | -1.04 | 2.0E-03 | -0.41 | 2.4E-01 |
| 1450650_at | *Myo10* | myosin X | -0.92 | 5.1E-04 | -1.35 | 2.0E-04 | -0.89 | 9.5E-04 |
| 1419105_at | *Nr1h4* | nuclear receptor subfamily 1, group H, member 4 | -0.87 | 2.7E-04 | -1.49 | 2.8E-05 | -1.35 | 2.0E-04 |
| 1438684_at | *Nuak1* | NUAK family, SNF1-like kinase, 1 | -0.97 | 1.2E-04 | -1.37 | 1.1E-03 | -0.57 | 3.7E-03 |
| 1422474_at | *Pde4b* | phosphodiesterase 4B, cAMP specific | -0.76 | 1.4E-04 | -1.32 | 4.6E-04 | -0.25 | 4.7E-02 |
| 1455185_s_at | *Phf16* | PHD finger protein 16 | -1.41 | 8.4E-06 | -1.10 | 3.0E-03 | -0.46 | 1.7E-02 |
| 1450060_at | *Pigr* | polymeric immunoglobulin receptor | -1.45 | 2.0E-02 | -1.08 | 4.7E-04 | -0.22 | 5.5E-01 |
| 1436821_at | *Plcxd3* | phosphatidylinositol-specific phospholipase C, X domain containing 3 | -0.84 | 9.2E-05 | -1.12 | 9.1E-04 | -0.22 | 2.0E-01 |
| 1453310_at | *Ppil6* | peptidylprolyl isomerase (cyclophilin)-like 6 | -1.39 | 1.1E-07 | -1.03 | 5.2E-04 | -0.68 | 6.8E-04 |
| 1435614_s_at | *Rasgrf1* | RAS protein-specific guanine nucleotide-releasing factor 1 | -1.05 | 1.1E-04 | -2.10 | 2.1E-03 | -1.98 | 1.4E-03 |
| 1434427_a_at | *Rnf157* | ring finger protein 157 | -0.73 | 6.7E-04 | -1.09 | 8.5E-06 | -0.30 | 5.4E-02 |
| 1427231_at | *Robo1* | roundabout homolog 1 (Drosophila) | -0.99 | 1.3E-05 | -1.17 | 1.1E-05 | -0.54 | 1.9E-03 |
| 1437784_at | *Runx1t1* | runt-related transcription factor 1; translocated to, 1 (cyclin D-related) | -0.53 | 1.7E-02 | -1.17 | 1.7E-04 | -0.37 | 5.3E-02 |
| 1457867_at | *Sgpp2* | sphingosine-1-phosphate phosphotase 2 | -0.78 | 1.7E-04 | -1.16 | 6.8E-05 | -0.59 | 8.8E-03 |
| 1423852_at | *Shisa2* | shisa homolog 2 (Xenopus laevis) | -1.70 | 5.2E-06 | -2.15 | 8.5E-05 | -0.57 | 7.1E-02 |
| 1418257_at | *Slc12a7* | solute carrier family 12, member 7 | -1.75 | 1.9E-08 | -1.40 | 6.1E-05 | -0.92 | 1.6E-02 |
| 1419656_at | *Slc25a36* | solute carrier family 25, member 36 | -0.62 | 3.7E-04 | -1.13 | 3.3E-05 | -0.66 | 4.2E-03 |
| 1455876_at | *Slc4a7* | solute carrier family 4, sodium bicarbonate cotransporter, member 7 | -1.01 | 1.2E-04 | -1.13 | 5.3E-05 | -0.36 | 2.6E-02 |
| 1455442_at | *Slc6a19* | solute carrier family 6 (neurotransmitter transporter), member 19 | -1.44 | 5.1E-06 | -1.11 | 1.7E-04 | -1.02 | 2.2E-04 |
| 1436555_at | *Slc7a2* | solute carrier family 7 (cationic amino acid transporter, y+ system), member | -0.67 | 1.7E-03 | -1.44 | 5.9E-05 | -0.63 | 7.8E-03 |
| 1451542_at | *Ssbp2* | single-stranded DNA binding protein 2 | -0.62 | 1.3E-04 | -1.27 | 1.5E-04 | -0.83 | 2.0E-03 |
| 1449409_at | *Sult1c2* | sulfotransferase family, cytosolic, 1C, member 2 | -1.82 | 1.6E-05 | -1.81 | 4.2E-06 | -1.47 | 1.1E-05 |
| 1448973_at | *Sult1d1* | sulfotransferase family 1D, member 1 | -2.07 | 5.7E-06 | -2.06 | 1.0E-05 | -1.79 | 1.4E-04 |
| 1429448_s_at | *Tet1* | tet oncogene 1 | -0.57 | 4.9E-03 | -1.34 | 3.7E-06 | -0.49 | 1.5E-03 |
| 1453360_a_at | *Tex9* | testis expressed gene 9 | -1.18 | 5.2E-04 | -1.78 | 1.3E-06 | -0.54 | 1.0E-01 |
| 1458347_s_at | *Tmprss2* | transmembrane protease, serine 2 | -0.73 | 9.0E-04 | -1.69 | 7.4E-06 | -0.36 | 1.0E-01 |
| 1426302_at | *Tmprss4* | transmembrane protease, serine 4 | -1.63 | 1.6E-06 | -1.48 | 4.2E-04 | -0.32 | 2.6E-01 |
| 1429870_at | *Tnik* | TRAF2 and NCK interacting kinase | -0.54 | 1.6E-04 | -1.01 | 1.1E-04 | -0.52 | 2.7E-03 |
| 1431530_a_at | *Tspan5* | tetraspanin 5 | -0.62 | 1.9E-03 | -1.15 | 1.0E-05 | -0.59 | 4.2E-03 |
| 1444214_at | *Tubb1* | tubulin, beta 1 | -1.32 | 4.9E-05 | -1.51 | 1.3E-04 | -1.32 | 3.3E-05 |
| 1427961_s_at | *Ugt2b34* | UDP glucuronosyltransferase 2 family, polypeptide B34 | -1.57 | 7.0E-04 | -1.56 | 2.8E-04 | -1.17 | 1.1E-04 |
| 1439624_at | *Ugt2b35* | UDP glucuronosyltransferase 2 family, polypeptide B35 | -0.94 | 1.2E-02 | -1.93 | 1.7E-07 | -1.69 | 1.8E-03 |
| 1419177_at | *Vps37a* | vacuolar protein sorting 37A (yeast) | -0.98 | 1.8E-03 | -1.16 | 2.5E-04 | -0.60 | 2.7E-03 |
| 1450055_at | *Vsnl1* | visinin-like 1 | -2.04 | 3.4E-08 | -1.69 | 3.0E-04 | -0.59 | 5.9E-03 |
| 1434170_at | *Wdr40b* | WD repeat domain 40B | -1.57 | 3.1E-06 | -1.43 | 1.4E-04 | -0.30 | 2.5E-01 |
| 1434401_at | *Zcchc2* | zinc finger, CCHC domain containing 2 | -0.71 | 3.3E-04 | -1.06 | 7.5E-06 | -0.21 | 1.2E-01 |
| 1441727_s_at | *Zfp467* | zinc finger protein 467 | -0.71 | 7.4E-03 | -1.03 | 2.0E-04 | -0.16 | 2.2E-01 |
| 1436153_a_at | *Zmynd11* | zinc finger, MYND domain containing 11 | -0.66 | 1.1E-03 | -1.07 | 1.5E-04 | -0.66 | 5.7E-03 |
