# Supplementary material for: Epistasis of Transcriptomes Reveals Synergism between Transcriptional Activators Hnf1α and Hnf4α
Source: PLoS Genet. 2010 May 27;6(5):e1000970. doi: 10.1371/journal.pgen.1000970 (PMC2877749; doi:10.1371/journal.pgen.1000970)
Supplement: Table S2 — Overrepresentation of functional classes among regulated genes in Hnf4a pKO and Hnf1a +/- islets. (0.03 MB PDF) [file pgen.1000970.s010.pdf]

SUPPL. TABLE 2 Overrepresentation of functional classes among regulated genes in *Hnf4a* ^{pKO} islets

**I. Downregulated Genes in Hnf4apKO islets**	
**Functional Annotation Clustering (DAVID 2008)**	
**Representative Terms in Cluster**	**Fisher Exact p value**
Transmembrane region	0.01
Selenium binding	0.02
Intercellular junction	0.03
Symporter activity, Ion transmembrane transporter activity	0.04
Sulfotransferase activity	0.04
Sugar transport	0.05
Signal transduction	0.06

**Gene Set Enrichment Analysis of Pathways**		
**Pathway**	**Nominal p value**	**FDR q value**
GHPATHWAY (BioCarta)	<0.0001	0.019
BIOSYNTHESIS_OF_STEROIDS (KEGG)	<0.0001	0.031
HSA01032_GLYCAN_STRUCTURES_DEGRADATION (KEGG)	<0.0001	0.042
HSA00450_SELENOAMINO_ACID_METABOLISM (KEGG)	<0.0001	0.114
ERK5PATHWAY (BioCarta)	<0.0001	0.131
WNTPATHWAY (BioCarta)	<0.0001	0.147
RARRXRPATHWAY (BioCarta)	<0.0001	0.196

**II. Upregulated Genes in *Hnf4a* ^{pKO} islets**	
**Functional Annotation Clustering (DAVID 2008)**	
**Representative Terms in Cluster**	**Fisher Exact p value**
Transmembrane region	0.01
Secreted; extracellular space	0.04
Lysosome	0.01
Cytokine activity	0.04
Polyamine metabolic process	0.02
MAPK signaling pathway	0.05
Cell adhesion	0.04
Response to stress	0.07
Extracellular matrix	0.10

**Gene Set Enrichment Analysis of Pathways**		
**Pathway**	**Nominal p value**	**FDR q value**
Up-regulated during TGFbeta-induced epithelial-to-mesenchymal transition (EMT)	<0.0001	<0.0001
MAPK_SIGNALING_PATHWAY (Kegg)	<0.0001	0.021
Upregulated by hypoxia in normal fibroblasts	<0.0001	<0.0001
TGF_BETA_SIGNALING_PATHWAY (Kegg)	<0.0001	0.023
REGULATION_OF_ACTIN_CYTOSKELETON (Kegg)	<0.0001	0.114
LEUKOCYTE_TRANSENDOTHELIAL_MIGRATION (Kegg)	<0.0001	0.002
INTEGRINPATHWAY (Biocarta)	<0.0001	0.001
TGF_BETA_SIGNALING_PATHWAY (Biocarta)	<0.0001	0.196
